# Supplementary material for: Measuring quality of DNA sequence data via degradation
Source: PLoS One. 2022 Aug 3;17(8):e0271970. doi: 10.1371/journal.pone.0271970 (PMC9348684; doi:10.1371/journal.pone.0271970)
Supplement: S1 Appendix — (PDF) [file pone.0271970.s001.pdf]

Background on data quality

This sketch draws on [1] and [2], with additional material from Office of Management and Budget (OMB) documents [3].

Data quality is a complex, multi-dimensional construct driven ultimately by data usage and subsequent decisions. There are two key questions: What are the intended uses of the data? What decisions are to be made based in part on the data? There are multiple hyperdimensions of data quality, each containing multiple dimensions:

Process Dimensions related to the generation, assembly, description and maintenance of data—reliability (with several sub-dimensions), metadata, security and confidentiality.[1]

Data Dimensions specifically associated with the data themselves. At the record/table level, these comprise accuracy, completeness, consistency and validity. Database-level dimensions are identifiability and joinability.

User Dimensions related to users and user—accessibility, integrability, interpretability, rectifiability, relevance and timeliness.

Objectivity Dimensions describing whether disseminated information is accurate, reliable, scientifically sounds and unbiased in terms of both substance and presentation.

Utility Dimensions addressing usefulness of the information for the intended audience's anticipated purposes.

Integrity Dimensions pertaining to protection of information from unauthorized, unanticipated or unintentional falsification or corruption.

Data quality cannot be disconnected from economics [5], because of the necessity to ask the question "To what end have the data been collected?" Data quality-associated costs are imposed on multiple classes of stakeholders, among them, data subjects, data collectors and stewards, decision makers and society at large. There are both actual and opportunity costs. Whether incurring them is justified in a particular cases depends on the associated decisions.

Because of the large, public financial consequences, the field in which data quality has received the most attention and deepest investigation is official statistics. The entire field of total survey error (TSE) has emerged in response [6, 7, 8], which is dominated by "total quality" thinking.

Measurement of data quality has long been a perplexing issue if construed narrowly to mean error rates,[2] because ground truth is unknown. In this paper, we applied clustering to identify outliers that may be—and in synthesized cases, *are*—data quality problems. In other settings,

[1] Modern industrial thinking about quality is, of course, dominated by focus on process rather than product [4]. Interestingly, and pertinent to this paper, the pioneer W. Edwards Deming spent a significant portion of his career at the U. S. Census Bureau. The increasingly important issue of data provenance also falls under process.

[2] In genomics, quality has historically focused solely on errors.

quality is measured by analytical utility. An example is statistical disclosure limitation, where data are altered deliberately in order to protect confidentiality, and there are quantifiable tradeoffs between disclosure risk and data quality. While sometimes problematic, because utility measures are often either too blunt or too narrow, this approach has been broadly productive.

Uncertainty has not dominated data quality strategies, but has lurked in the background for years. Recent approaches such as "fitness for purpose" [9] and a "decision quality rather than data quality" focus accounts implicitly for uncertainty. Re-formulated as "Total Survey Uncertainty," total survey error (TSE) can address uncertainty. Yet another means of accommodating uncertainties is explicit consideration of risk [10].

References

[1] Karr AF, Sanil AP, Banks DL. Data quality: A statistical perspective. Statistical Methodology. 2006;3(2):137–173.

[2] Karr AF. Discussion of five papers on "Systems and Architectures for High-Quality Statistics Production". Journal of Official Statistics. 2013;29(1):157–163.

[3] Office of Management and Budget. Standards and Guidelines for Statistical Surveys; Statistical Policy Directive No. 2; 2006.

[4] Deming WE. Quality, Productivity, and Competitive Position. Cambridge, MA: Massachusetts Institute of Technology, Center for Advanced Engineering Study; 1982.

[5] English LP. Improving Data Warehouse and Business Information Quality: Methods for Reducing Costs and Increasing Profits. New York: Wiley; 1999.

[6] Biemer PP, Lyberg LE. Introduction to Survey Quality. Hoboken, NJ: John Wiley & Sons, Inc.; 2003.

[7] Groves RM. Survey Errors and Survey Costs. New York: Wiley; 2004.

[8] Biemer PP, Leeuw ED, Eckman S, Edwards B, Kreuter F, Lyberg L, et al., editors. Total Survey Error in Practice. New York: Wiley; 2017.

[9] Mocnik FB, Zipf A, Fan H. Data quality and fitness for purpose; 2017. Available from: `https://www.researchgate.net/publication/316829634`.

[10] Eltinge JL, Biemer PP, Holmberg A. A Potential Framework for Integration of Architecture and Methodology to Improve Statistical Production Systems. Journal of Official Statistics. 2013;29:1:125–145.
