# Supplementary material for: Measuring quality of DNA sequence data via degradation
Source: PLoS One. 2022 Aug 3;17(8):e0271970. doi: 10.1371/journal.pone.0271970 (PMC9348684; doi:10.1371/journal.pone.0271970)
Supplement: Attachment — Submitted filename: RebuttalLetter.pdf [file pone.0271970.s002.pdf]

Rebuttal Letter

May 23, 2022

We thank the reviewers and editors for their comments. Specific responses are as follows:

Formatting The entire manuscript has been converted to the PLOSOne LaTeX template, with the correct section titles and the bibliography entries included in the source file `DataQualityViaDataDegradation_PLOSOne_Revised.tex`. Footnotes have been eliminated. The previous appendix has been converted supplementary material, namely S1 Appendix. Because of the scope of the changes and use of LaTeX, no "tracked changes" file can sensibly be created.

Figures Because figure numbering has changed, a completely new set of TIFF files has been uploaded.

Self-referencing has been reduced dramatically.

Funding Statement Funding information has been removed from the manuscript. No changes are necessary to the Funding Statement.

Data availability Data files are now publicly available at `https://dataverse.harvard.edu/dataset.xhtml ?persistentId=doi%3A10.7910%2FDVN%2FQ6HVFO&version=DRAFT`.

ORCID ID I believe that I have entered it successfully. However, in case there are any issues, it is 0000-0002-7253-0129, and I give my permission to add it. ORCID IDs for all authors who have them appear in the manuscript.
